# Effect of Probiotics on Stress Scores, Cortisol Levels, and Immune Functions in Moderately Stressed Adolescents and Young Adults: Results of a Feasibility Clinical Study

**DOI:** 10.3390/ph19101662

**Published:** 2026-10-21

**Authors:** Elena Y. Enioutina, Manav Jain, Rachel Hudson, Brian Bradford, Kevin M. Watt, Nicole L. Mihalopoulos, Simon Brewer

**Affiliations:** 1 Pediatrics Department, Spencer Fox Eccles School of Medicine, University of Utah, Salt Lake City, UT 84108, USA; jain.manav@ufl.edu (M.J.); rachel.e.hudson0217@gmail.com (R.H.); kevin.watt@hsc.utah.edu (K.M.W.); nikkislc@gmail.com (N.L.M.); 2 Psychiatry Department, Spencer Fox Eccles School of Medicine, University of Utah, Salt Lake City, UT 84108, USA; 3 School of Environment, Society & Sustainability, University of Utah, Salt Lake City, UT 84112, USA; simon.brewer@ess.utah.edu

**Keywords:** probiotics, adolescents, young adults, stress, secretory immunoglobulin A, salivary cytokines

## Abstract

**Background:** Stress can negatively affect immune system functions by regulating cytokine production. The purpose of this feasibility study was to investigate the effects of a probiotic supplement on stress, cortisol levels, and the immune functions of moderately stressed adolescents and young adults. **Methods:** A randomized, single-blind clinical trial was conducted at Primary Children’s Hospital, Utah. Eighty-seven moderately stressed (Perceived Stress Score, PSS, 14–26) volunteers (12–20 years old) completed the study. Participants took either a probiotic blend containing three *Lactobacillaceae* strains or placebo capsules for 12 weeks. Participants’ PSSs were evaluated following office visits at baseline, and after 8 and 12 weeks of supplementation. Participants’ collected salivary samples were analyzed for cortisol, secretory IgA, and a panel of cytokines (IL-1β, IL-6, IL-8, TNF-α, and IL-10). **Results:** Both cohorts showed decreases in PSS and increases in morning salivary cortisol levels; the difference between cohorts was not statistically significant. Probiotics slowed the increase in cortisol levels and had a beneficial effect on cytokine production, with a statistically significant reduction in IL-1β levels compared to placebo (*p* = 0.03–0.004). A statistically significant, weak positive correlation between cortisol levels and IL-1β (r = 0.16, *p* = 0.015) has been observed. **Conclusions:** This study’s results suggest that probiotic supplementation may influence cortisol dynamics and cytokine responses in moderately stressed adolescents and young adults, with IL-1β showing the most consistent between-cohort difference. These findings support further investigation of probiotic supplementation in more rigorously designed trials that account for the types of stress experienced by participants in early life and during the study.

## 1. Introduction

Stress is a physiological and emotional reaction to challenging or perceived threatening situations [1]. The most common stress that people may experience on an everyday basis is acute stress, a short-term reaction to a perceived threat or challenge. Chronic stress, however, is a long-term feeling of being overwhelmed, presenting with insomnia, fatigue, irritability, depression, and anxiety. Adolescents and young adults are exposed to an array of stressors daily [2,3]. This might be a heavy academic workload, the need to meet assignment deadlines, bullying, or interpersonal relationships with friends, family members, or neighbors [3]. More than 30% of children and adolescents may experience stress-related health problems, presented as abdominal or chest pain, headaches, and some other conditions [3,4].

Cortisol is one of the major regulators of the human reaction to stress [5,6]. Its secretion is initiated upon perception of threat through a cascade of hormones secreted in the hypothalamic–pituitary–adrenal (HPA) axis, a primary endocrine stress response system [5].

Psychological stress may also affect immune functions. A meta-analysis of 300 studies investigating the relationship between stress and immune functions revealed the differential effects of acute and chronic stresses on immune cell functions [7]. Several studies reported increases in circulating plasma cytokines, including IL-6, IL-1β, TNF-α, and IL-10, in response to acute psychological stress in adults [8,9,10]. Acute psychological stress has stimulated a rapid increase in salivary IL-6 and IL-1β but not TNF-α in 18–30-year-old healthy men [11]. Another study showed that academic examination significantly upregulated the production of TNF-α, IL-6, IFN-γ, and IL-10 in medical students [12]. Students who had higher perceived stress scores (PSSs) had higher levels of inflammatory cytokines. Adolescents exposed to early-life adversity showed an increased expression of cytokine and innate anti-viral defense gene transcripts [13]. On the contrary, it was reported that in adolescents (12–19 years old), stress induced by a public speaking task did not affect cytokine production [14]. Chronic stress may differentially affect adolescents who were exposed to childhood adversity compared to those who were not [15]. Adolescents who were not exposed to childhood adversity but had recent chronic stress had lower expression of inflammatory cytokine genes.

Probiotics are living microorganisms that positively affect microbiomes, support functions of numerous organs and systems, and overall human health [16,17]. Probiotics demonstrate immunomodulatory effects on pro- and anti-inflammatory cytokines [18]. Probiotics may help to balance stress levels by regulating signaling through the gut–brain axis [19]. Additionally, probiotics may ameliorate stress and depression symptoms by decreasing the production of pro-inflammatory cytokines [20]. Elevated cortisol levels in stressed individuals increase intestinal barrier permeability and the production of inflammatory cytokines by bacteria-activated immune cells. In mouse models of stress, probiotics decreased corticosterone levels and restored the gut barrier.

The purpose of this randomized, single-blind feasibility clinical study was to investigate the effects of a probiotic blend comprising two *Lactiplantibacillus plantarum* strains and *Levilactobacillus brevis* on PSS, morning salivary cortisol, secretory IgA, and cytokine levels in adolescents and young adults experiencing moderate stress. This feasibility study would help to identify potential problems with study design.

## 2. Results

### 2.1. Demographic Characteristics of Participants

**Figure 1:**
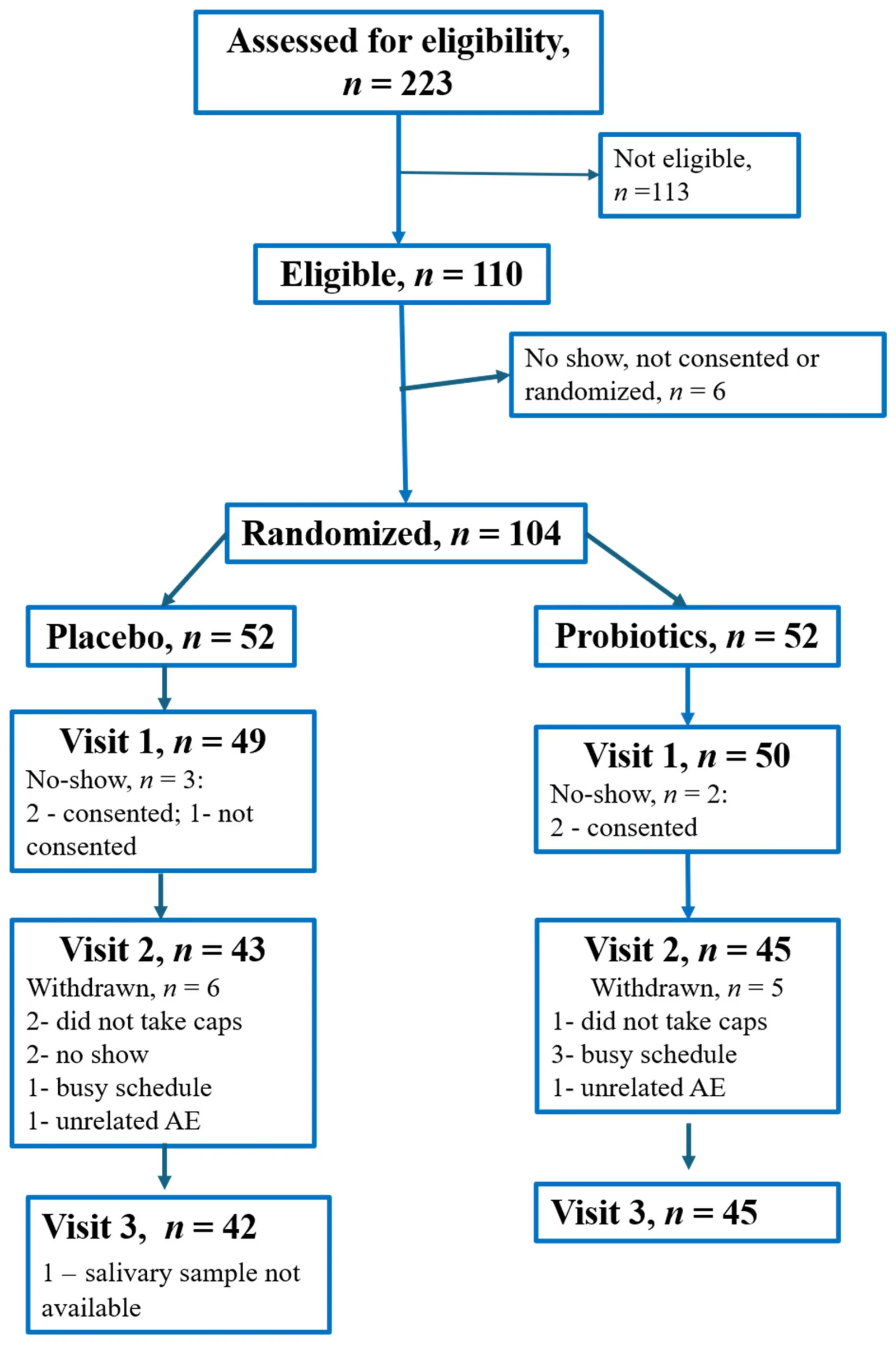
Study flowchart.

The study was advertised at Primary Children’s Hospital, Utah Urban School Districts, and on social media during the 2022–2023 and 2023–2024 school years. Potential participants, their parents or guardians contacted the study coordinators via study-designated email. Of 223 potential participants, ~51% were not eligible: (a) individuals with average PSS less than 14 or greater than 26 and (b) individuals receiving selective serotonin reuptake inhibitors (SSRIs). Of 110 eligible individuals, 6 decided not to participate in the study after pre-screening. One hundred and four participants were randomized to placebo (*n* = 52) and probiotics (*n* = 52) cohorts (Figure 1). Of 99 participants who attended visit 1 (baseline assessment), 87 participants (88%) completed the study. The main reasons for self-withdrawal were: (a) parents or guardians reported that a participant had not taken probiotic capsules and withdrew the participant; (b) participants had busy schedules and could not attend in-person visits.

No serious adverse events were reported in this study. One participant reported nausea and occasional vomiting after taking probiotic capsules in the first two weeks of the study. The participant considered this normal when taking probiotics and reported it on visit 2. Two participants had unrelated adverse events.

The demographic characteristics of the participants who completed the study are presented in Table 1. Most of the enrolled participants were non-Hispanic White. Approximately half of the participants were females. The average age of participants was 14.7 and 15.2 years in the placebo and probiotic cohorts, respectively. The average stress scores were comparable between the cohorts.

### 2.2. Stress Scores and Cortisol Levels

**Figure 2:**
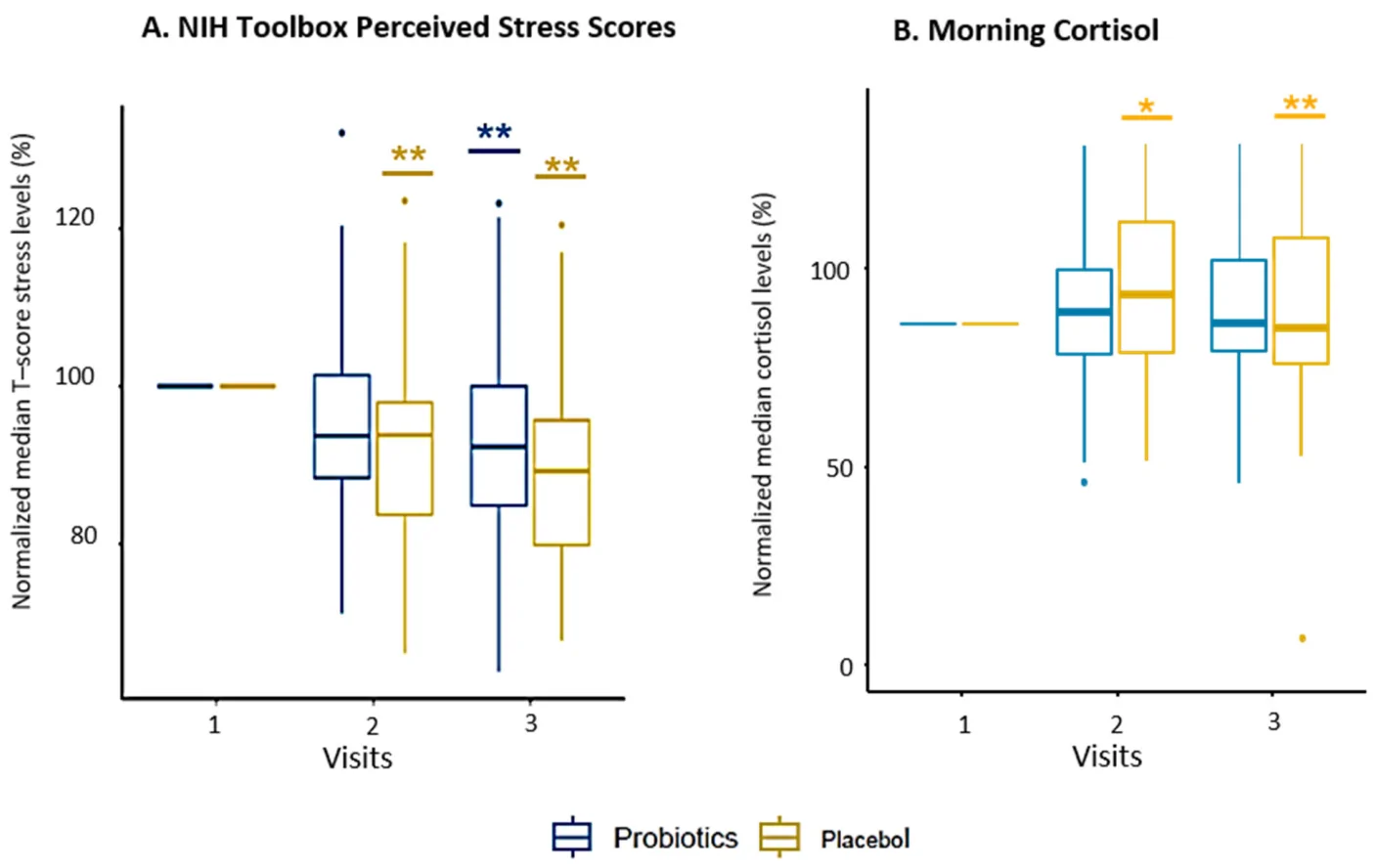
Normalized PSS and morning cortisol levels. The median levels were normalized to baseline (visit 1). The boxplot shows the first (Q1) and third (Q3) quartiles, the median, and outliers indicated as blue dots for the probiotic and yellow dots for placebo cohorts. (**A**) Normalized median T-scores, (**B**) normalized morning cortisol levels. *—within-cohort differences were statistically significant, *p*-value < 0.05; **—within-cohort differences were statistically significant, *p*-value < 0.005.

Stress scores of eligible participants were assessed using the NIH Toolbox Perceived Stress FF Ages 13–17 v2.0 and the NIH Toolbox Perceived Stress FF Ages 18+ v2.0 during office visits 1, 2, and 3. This fixed-form (FF) assessment measures participants’ perceptions of how unpredictable, uncontrollable, and overloaded their lives are. T-scores adjusted for age and gender were used in the study. Due to high variation in T-scores between participants in cohorts, median T-scores (hereafter, PSS) were normalized to baseline (visit 1, 100%). The normalized median PSS declined during the study period in both cohorts (Figure 2A). Within-cohort differences relative to baseline were statistically significant. In the probiotic cohort, the *p*-value between baseline and visit 3 was 0.0006; in the placebo cohort, the *p*-value between baseline and visit 2 was 0.001, and between baseline and visit 3 was 0.0001. However, differences between the probiotic and placebo cohorts were not significant. We further investigated correlations of PSS with other covariates. A significant positive correlation was found between age and PSS (r = 0.23, *p* < 0.001), but no gender-specific differences were observed (W = 1.53, *p* = 0.127).

Since stress is associated with elevated cortisol levels, this study also measured cortisol. Salivary samples were collected by participants in the morning (before 8:00 AM). Most participants collected their morning samples before 8:00 AM, with some exceptions on weekends. An increase of 20% in the normalized median morning cortisol levels was observed in the probiotic cohort in visit 2 samples compared to baseline; then, cortisol levels declined and exceeded baseline by only 3% (Figure 2B). The differences between visits 2 and 3 and baseline in normalized median cortisol levels were not statistically significant in the probiotic cohort (visit 2: *p* = 0.055; visit 3: *p* = 0.071). An increase in median cortisol levels was also observed in the placebo cohort. It increased by 56% in the visit 2 samples and 7% in the visit 3 samples compared with baseline. Within-cohort differences in the placebo cohort were statistically significant (visit 2: *p* = 0.02; visit 3: *p* = 0.003). Differences between cohorts were not statistically significant at any time point. No correlation was observed between normalized median cortisol levels and PSS in either the probiotic (r = 0.03, *p* = 0.745) or placebo (r = −0.17, *p* = 0.066) cohorts. Additionally, no evidence was found for gender-specific changes in stress scores or cortisol levels.

### 2.3. Immune Biomarkers

Salivary samples were analyzed for secretory IgA (sIgA) and cytokine levels. There were significant variations in analyte levels in participants’ samples. The baseline median levels of IL-6 and IL-8 between probiotics and placebo cohorts were statistically different (IL-6: *p* = 0.04; IL-8: *p* = 0.04). Due to significant variation in sIgA and cytokine levels, the median levels were normalized to 100% of baseline (for details, see Section 4.5).

#### 2.3.1. Secretory IgA

**Figure 3:**
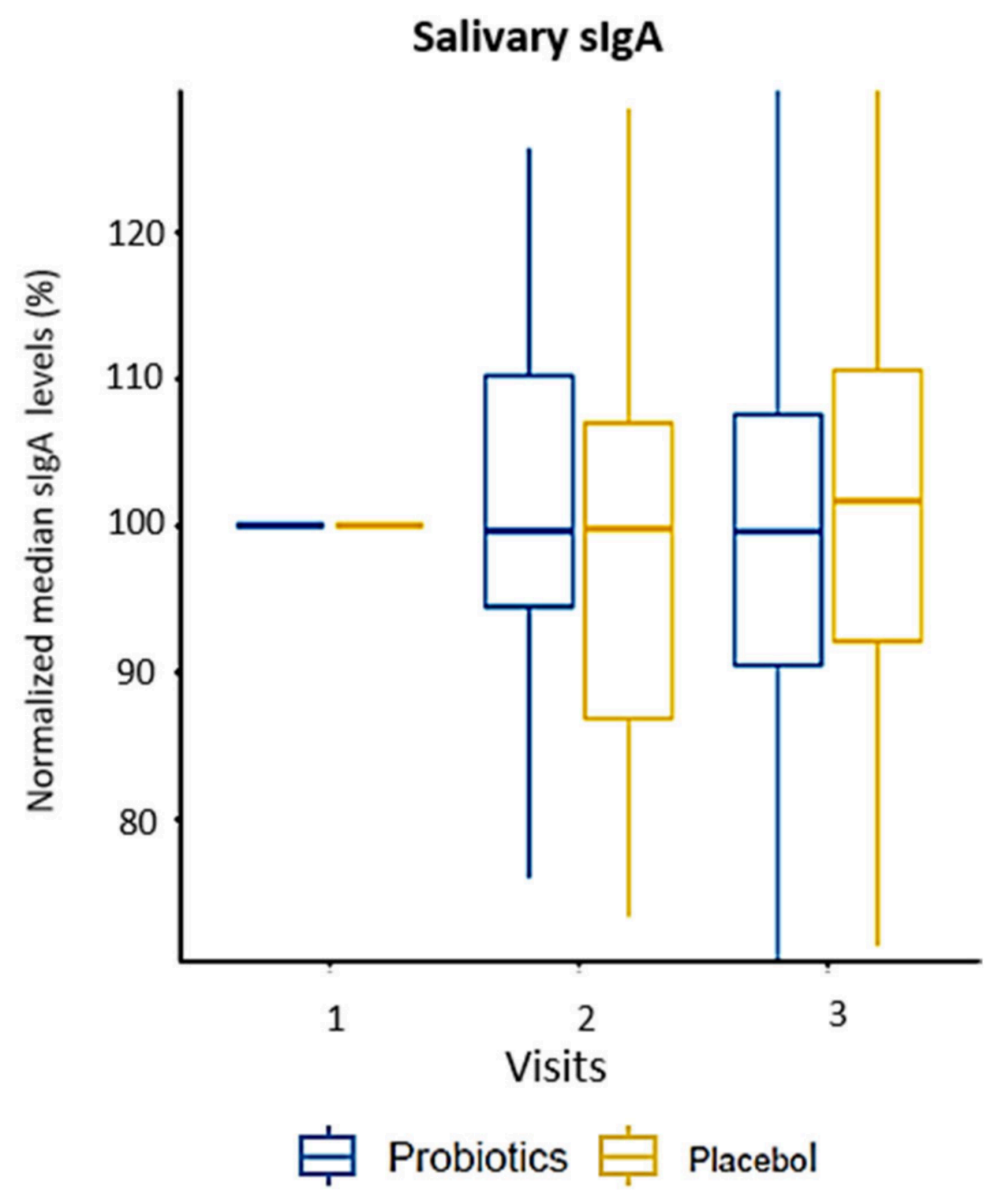
Normalized salivary sIgA levels. The median sIgA levels were normalized to baseline (visit 1). The boxplot shows the first (Q1) and third (Q3) quartiles, the median, and outliers. Within-cohort and between-cohort differences were not statistically significant.

The salivary sIgA levels did not change significantly during the study (Figure 3). In both cohorts, sIgA-normalized median levels slightly decreased (−1%) in samples from participants in both cohorts at visit 2. The sIgA levels remained the same in the visit 3 samples from the probiotic cohort, while sIgA levels in the placebo cohort samples slightly increased compared with baseline. Within-cohort and between-cohort differences were not statistically significant.

#### 2.3.2. Salivary Cytokine Levels

**Figure 4:**
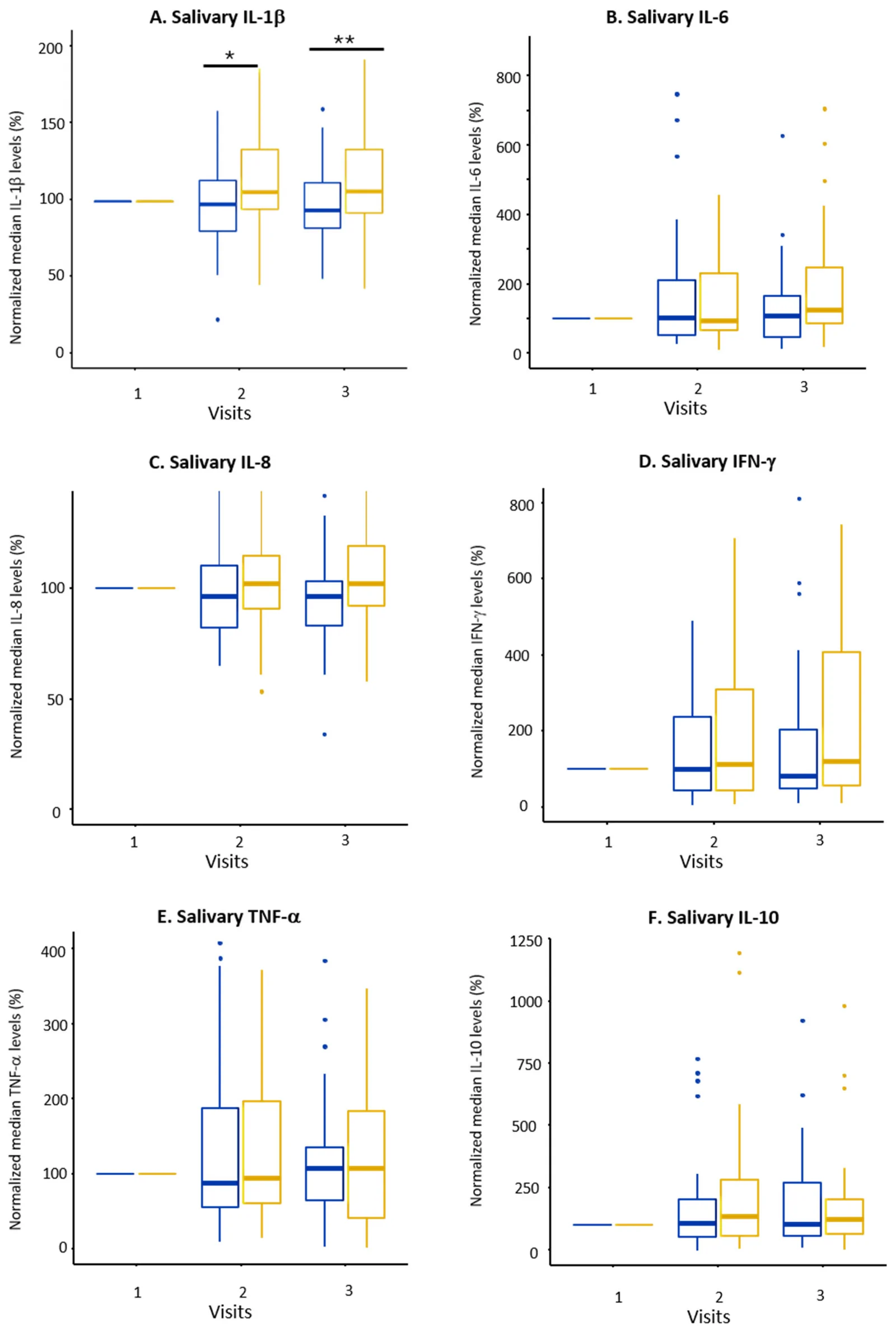
Normalized salivary cytokine levels. The median cytokine levels were normalized to baseline (visit 1). The boxplot shows the first (Q1) and third (Q3) quartiles, the median, and outliers indicated as blue dots for the probiotic and yellow dots for placebo cohorts. (**A**)—IL-1β; (**B**)—IL-6; (**C**)—IL-8; (**D**)—IFN-γ; (**E**)—TNF-α; (**F**)—IL-10. The differences in normalized IL-1β levels between placebo and probiotic cohorts were statistically significant; *—*p* = 0.03 and **—*p* = 0.004.

Normalized median levels of IL-1β decreased by 2% in visit 2 samples and 5.5% in visit 3 samples compared to the baseline in the probiotic cohort (Figure 4A). Within-cohort differences were not statistically significant. The use of placebo capsules increased the normalized median IL-1β levels by 6–7% compared to baseline during the study. Within-cohort differences were statistically significant compared to baseline; *p*-values were 0.03 for visit 2 samples and 0.001 for visit 3 samples. Differences in normalized IL-1β levels between placebo and probiotic cohorts were statistically significant at visit 2 (*p* = 0.03) and at visit 3 (*p* = 0.004). Correlation between PSS and normalized median IL-1β levels showed a statistically significant, negative relationship (r = −0.25, *p* = 0.005) for participants receiving placebo but this was not significant for participants receiving probiotics (r = 0.11, *p* = 0.242). Nonetheless, there was a statistically significant, weak positive correlation between cortisol levels and IL-1β (r = 0.16, *p* = 0.015). No correlation was found between IL-1β and age (*p* = 0.203) or between IL-1β and gender (*p* = 0.11) when intra-subject variability was accounted for.

The normalized median IL-6 levels decreased by 53% (visit 2) and 27.3% (visit 3) compared to baseline in the probiotic cohort (Figure 4B). There was a decrease of 10.7% in the normalized median IL-6 levels in the placebo cohort compared to baseline in visit 2 samples, followed by an increase of 15.2%. Due to significant variation in the IL-6 levels in both cohorts, the differences between the cohorts were not statistically significant.

The IL-8 levels were highly variable in both cohorts. In the probiotic cohort, the normalized median levels of IL-8 decreased by ~3% in visit 2 and visit 3 samples compared to baseline (Figure 4C). The IL-8 levels in the placebo cohort increased by 2.5% compared to baseline. The within-cohort changes were not statistically significant in either cohort. The differences in the normalized median IL-8 levels at visits 2 and 3 compared to baseline between the placebo and probiotic cohorts were also not statistically significant.

There was a decrease in normalized median IFN-γ levels in both cohorts, ranging from 42.7% to 67.2% (Figure 4D). The within-cohort and between-cohort differences in the IFN-γ levels were not statistically significant. The normalized median TNF-α levels decreased by 15–48% with placebo and by 19–24% with probiotics during the study compared to baseline (Figure 4E). All within-cohort and between-cohort differences were not statistically significant.

There was a decrease in the normalized median IL-10 levels compared to baseline by 68% in the visit 2 salivary samples and by 49.5% in the visit 3 samples compared to baseline samples in the probiotic cohort (Figure 4F). In the placebo cohort, normalized median levels also decreased by 45% on visit 2 and 30% on visit 3 compared to baseline. The within- and between-cohort differences were not statistically significant.

## 3. Discussion

One of the primary outcomes of this feasibility study was the evaluation of the effect of probiotics on PSS levels among moderately stressed adolescents and young adults attending high school during the study. The study participants were exposed to a variety of new stressors (e.g., new classmates, new teachers, assignments, and exam deadlines) during the study. Both probiotic and placebo cohorts showed a statistically significant decrease in PSS levels. Potentially, the reduction in PSS levels can be explained by the favorable adaptation of some participants to stressful situations during the school year [21].

Elevated cortisol levels are objective indicators of stress experienced by individuals. Cortisol levels fluctuate in healthy individuals based on circadian rhythms [22]. Typically, cortisol levels are highest in the early morning and lowest at night. The HPA axis is an essential stress response system that controls cortisol production [23]. Chronic stress may stimulate prolonged release of cortisol, resulting in blunting of HPA axis responses [22]. Emotion regulation strategies (e.g., suppression and reappraisal) induced by acute stress may amplify heightened cortisol activity [24]. Although we did not observe statistically significant differences in morning cortisol levels between placebo and probiotic cohorts, probiotic use slowed the rise in cortisol levels (20% increase in visit 2 samples) compared to baseline. In the placebo cohort, however, cortisol levels increased by 56% in visit 2 samples compared to baseline. This might suggest a beneficial effect of the probiotic blend on the regulation of HPA axis responses to stress, resulting in lower cortisol levels. There was no statistically significant correlation between PSS and cortisol levels. This finding is in agreement with the results of another study demonstrating no correlation between salivary “Cortisol Awakening Response” and PSS in adolescents [25].

Recent studies confirmed that the use of prebiotics and probiotics improved psychological scores of people predisposed to mood disorders [26]. A triple-blind, placebo-controlled, randomized clinical trial showed that supplementation of the diet of healthy volunteers with a probiotic for four weeks resulted in a significant reduction in subjects’ rumination and aggressive thoughts [27]. Chong and colleagues studied the ability of the probiotic, *Lactiplantibacillus plantarum* DR7, to reduce signs of stress and improve immune function in adults [28]. The authors reported that 12-week consumption of the probiotic reduced signs of stress and improved participants’ psychological scores. Use of this probiotic led to a reduction in plasma cortisol levels.

Stress and elevated cortisol levels may affect the immune functions of people [21]. Monocytes obtained from people experiencing physiological stress exhibited an increased inflammatory profile upon stimulation through Toll-like receptors [29]. Salivary IL-6 and IL-1β were increased in healthy young men undergoing the Trier Social Stress Test [11]. Academic examination of medical students resulted in increased levels of TNF-α, IL-6, IL1Ra, IFN-γ, and IL-10 [12]. Importantly, students with high anxiety responses had higher levels of IFN-γ and lower levels of anti-inflammatory IL-10 and IL-4 cytokines. Chronic stress stimulated IL-1β production [22]. IL-1β levels may be upregulated in situations when people cannot “deal” with stressful situations. The use of *Lactiplantibacillus plantarum DR7* reduced levels of inflammatory cytokines (e.g., IFNγ) [28]. *Lactobacillaceae* species produce soluble poly-P granules, and their production may also be associated with enhanced barrier function of gut epithelial cells and reduced inflammation [30]. The results of our study showed that the probiotic blend containing *Lactiplantibacillus plantarum* and *Levilactobacillus brevis* strains modified salivary levels of IL-1β, IL-6, IL-8, TNF-α, IFN-γ, and IL-10. The differences were not statistically significant compared to the placebo cohort except for IL-1β. The probiotic use mainly affected salivary IL-1β levels. Salivary IL-1β levels decreased in the probiotic cohort, while levels of IL-1β in the placebo cohort rose during the study. The negative correlation between PSS and normalized median IL-1β levels was significant in participants receiving placebo but not significant in those receiving the probiotics. It appears that the use of probiotics disrupted this negative correlation. There is no clear explanation for the negative correlation between PSS and IL-1β in the placebo cohort of adolescents and young adults. To confirm or disprove this observation, a larger-scale study is necessary.

PSS is a reliable but more subjective measurement of stress levels, especially in adolescents. Cortisol is another biomarker of stress. We found a statistically significant weak positive correlation between cortisol levels and IL-1β. In adults experiencing panic disorders, there was a negative correlation between cytokines IL-1β and IL-12 and cortisol levels [31]. There was a similar trend as with IL-1β when IL-8 levels decreased in the probiotic cohort and increased in the placebo cohort during the study. Levels of IL-6 decreased in both cohorts. However, the magnitude of decrease was much higher in the probiotic cohort (53% vs 10.7% at visit 2).

The effects of probiotics may depend on the route of administration [32]. It has been shown that “local” administration of probiotics increased salivary IL-1β but not salivary IL-6 levels. This may be explained by oral cavity immune cells’ reaction to the introduction of probiotic bacteria to the oral cavity. *Lactiplantibacillus plantarum* can modulate immune responses through activation or suppression of macrophage and T cell activities [33]. *Lactiplantibacillus plantarum* suppressed inflammatory cytokines (e.g., IL-1β, IL-6, and IFN-γ) and stimulated anti-inflammatory cytokines (IL-10) in mice [34]. Both *Lactiplantibacillus plantarum* and *Levilactobacillus brevis* attenuated LPS-induced nuclear factor-κB (NF-κB) nuclear translocation [35]. *Bifidobacterium longum* subsp. *infantis* suppressed inflammatory and stimulated anti-inflammatory responses via production of exopolysaccharides [36].

Our finding that oral use of probiotics did not affect sIgA production is in good agreement with the findings of a systematic review [32].

Most studies investigating stress or stress and probiotics effects on the immune system were conducted in adult participants. The reaction of children and adolescents to stressors and probiotics can be different due to early-life adversity events experienced by participants [13]. Our study did not collect information regarding early-life adverse events and recent stressors experienced by the study participants.

Additionally, our participants collected salivary samples at home. Varying the time of sample collection and storage conditions may contribute to significant variability in cortisol and cytokine levels.

## 4. Materials and Methods

### 4.1. Study Product

The dietary supplement used in this study is a blend of probiotics: *Lactiplantibacillus plantarum* CECT7481 (formerly *Lactobacillus plantarum* CECT 7481, *Levilactobacillus brevis* CECT7480 (formerly *Lactobacillus brevis* CECT7480), and *Lactiplantibacillus plantarum* CECT7485 (formerly *Lactobacillus plantarum* CECT7485). One capsule of the blend contained 2 billion CFU of probiotic blend in a ratio of 2 (*Lactiplantibacillus plantarum* CECT 7481):1 (*Levilactobacillus brevis* CECT7480):1 *Lactiplantibacillus plantarum* CECT7485. Placebo and probiotic capsules were provided by Kaneka North America LLC (Pasadena, TX, USA). The capsules were stored in a −20 °C refrigerator before being distributed to the participants. The potency of the probiotic blend was evaluated at the beginning of the study and throughout the study period by a third-party laboratory.

### 4.2. Inclusion/Exclusion Criteria

Inclusion Criteria: Eligible volunteer participants of the study were:

1. Participants, males and females.
2. Participants who are 12–20 years old.
3. Participants with sustained moderate stress levels as determined by the Perceived Stress Scale 10 (PSS-10, [37]), with average scores of 14–26 on two pre-screening measures one week apart.
4. Participants with BMI percentile >5th% and <97th%.
5. Participants receiving or not receiving cognitive behavioral therapy.

Exclusion Criteria:

1. Participants who are less than 12 years of age or over 20 years of age at enrollment.
2. Participants with PSS average scores less than 14 and more than 26.
3. Participants with BMI percentile <5th% or >97th%.
4. Participants receiving selective serotonin reuptake inhibitors (SSRIs).
5. Participants who take any nutraceutical affecting stress or anxiety within one month before subject screening.
6. Participants who are pregnant.
7. Participants receiving immunosuppressive therapy
8. Participants with immune deficiencies
9. Participants who had adverse reactions to probiotics in the past.

### 4.3. Study Objectives

This was a feasibility study intended to characterize the effect of a probiotic dietary supplement on perceived stress levels, morning salivary cortisol, and the salivary immune responses of moderately stressed adolescents and young adults.

### 4.4. Study Design

The randomized, feasibility, single-blind (participants) clinical study was conducted by the University of Utah Pediatrics Department at Primary Children’s Hospital in 2022–2024. Ethics approval was obtained from the University of Utah Institutional Review Board (IRB, No. 00149912). The study was registered at clinicaltrials.gov (registration number NCT 07565883). The study was advertised in Salt Lake and Jordan District high schools using printed study flyers or information about the study sent to students, parents, and caregivers through the school networks.

#### 4.4.1. Pre-Screening Visit

The pre-screening process included two short Zoom^®^ or telephone interviews within two weeks to determine the potential participant’s stress levels and to ensure that the participant meets the inclusion/exclusion criteria. One of the inclusion criteria is sustained moderate stress levels (PSS 14–26) measured by the PSS-10 test [37]. To ensure the substantivity of stress levels in potential participants, the PSS tests were administered two times before participants were consented. Participants and their parents were offered an option to ask questions about the study during Zoom^®^/phone interviews or to schedule an additional Zoom^®^ meeting or phone call during which PIs or the study team member could answer questions about the study. If participants were eligible to participate in the study, their parents were asked to sign a parental permission form, and the adolescents were asked to sign an assent form. If potential participants were 18–20 years old, they signed an informed consent.

#### 4.4.2. Randomization and Sample Size Rationale

Participants were randomly assigned to the probiotic or placebo cohort using randomizer.org. The target enrollment was 55 participants in the probiotic cohort and 55 participants in the placebo cohort, with a rationale to have more than 30 participants in each cohort who completed the study [38].

#### 4.4.3. Participant Instructions for Capsule Use

The participants started taking capsules the day after the first visit, after collecting baseline saliva samples, and continued taking them daily (preferably with dinner) for 12 consecutive weeks. If the probiotic was not taken during dinner, it was recommended to be taken within 2 h after dinner or with the next meal.

#### 4.4.4. Screening Visits

All participants who completed the study had three in-person visits to evaluate their stress levels at Primary Children’s Hospital: a baseline visit (visit 1), an 8-week visit (visit 2), and a 12-week visit (visit 3) (Table 2). At office visit 1, participants were supplied with white, unlabeled boxes containing placebo or probiotic capsules. Placebo and probiotic capsules looked identical; both were off-white. Each participant was verbally instructed on how to take the capsules and collect saliva. Additionally, the participants received written/pictogram instructions, which they could place in their rooms.

During office visits, stress scores of participants were analyzed using the NIH Toolbox Perceived Stress FF v2.0 and calculated as a T-score adjusted for age and gender [39]. This was a fixed-form (FF) assessment of the participant’s perceptions of how unpredictable, uncontrollable, and overloaded their lives are.

Participants were asked to bring blisters with unused capsules to visits 2 and 3. Information about unused capsules was added to participation records. Typically, participants did not take 2–4 capsules over 12 weeks. Three participants did not take capsules regularly (Figure 1). Parents reported this to the PI. Participants were withdrawn from the study by the PI.

Salivary samples were collected by participants at their homes using sample collection kits (Salimetrics, Carlsbad, CA, USA). Pre-labeled cryovials and saliva collection kits were given to the participants at visit 1. Participants were asked to collect salivary samples in the morning before 8:00 AM the next day after visit 1 and 1–2 days before coming to visits 2 and 3. Saliva collection into pre-labeled cryovials was guided by the OnTimePoint™ Saliva Collection Management App, Salimetrics LLC (Carlsbad, CA, USA). Samples were stored in the participants’ freezers. On office visit days, participants were asked to bring samples to Primary Children’s Hospital on ice to ensure they remained frozen. The study coordinator or PI collected the samples and placed them in the −80 °C freezer. Coded samples were stored frozen at −80 °C until shipment to Salimetrics for cortisol, cytokine (IL-1β, IL-6, IL-8, TNF-α, IFN-γ, and IL-10), and sIgA analysis. All samples were analyzed in duplicate.

#### 4.4.5. Study Outcomes

The primary outcomes of this study focused on evaluating stress and cortisol levels in moderately stressed adolescents and young adults. Additionally, the effects of a probiotic dietary supplement on participants’ salivary inflammatory/anti-inflammatory cytokine and sIgA production were investigated.

### 4.5. Statistical Analysis

Pairwise comparisons were evaluated using the Wilcoxon rank-sum test for continuous variables and the chi-squared test or Fisher’s exact test for categorical variables. Bivariate correlations of continuous variables were assessed using Pearson’s correlation coefficient. All *p*-values reported are corrected for multiple testing using Holm’s method [40].

All test results (cytokines, sIgA, and NIH toolbox PSS) were visually inspected using boxplots and parallel coordinate plots. Normality checks were performed using the Shapiro–Wilk test for values from each visit for probiotics and placebo cohorts, respectively. To allow interpretation of changes as percentages from visit 1 (baseline) and to remove any baseline differences between the treatment and placebo cohorts, all values were log-transformed, then normalized per individual as the ratio between the value at a given visit and the value at visit 1.

\hat{y} = \frac{y_i}{y_1} ∗ 100

where y_i is the concentration at a given visit, and y_1 is the value at visit 1. Median percent changes are reported.

Changes in normalized toolbox scores or Salimetrics concentrations were then compared between placebo and probiotics and across the three visits, using a linear mixed-effect model that was fitted using the lme4 package [41] in R 4.5.1 [42] based on the following equation:

\hat{y}_ij = β_1 + γ_1 (Control = 1) + β_2 (Time = 2) + γ_2 (Control = 1:Time = 2) + β_3 (Time = 3) + γ_3 (Control = 1:Time = 3) + u_j + e_ij

where β_1 is the expected value for the treatment group at time 1 (before treatment); γ_1 is the difference in the control group at time 1; β_2 is the change in the treatment group from time 1 to time 2; γ_2 is the difference between control group and the treated group at time 2; β_3 is the change in the treatment group at from time 1 to time 3; γ_3 is the difference between control group and treated group at time 3. u_j represents the random effect for participant j, and e_ij represents the residual error, assumed to be normally distributed. Post hoc pairwise comparisons were then estimated using the emmeans package [43] to compare changes between visits for cases and controls, and between cases and controls for each visit. Outliers exceeding 2 standard deviations were removed from the graphs to better represent the median levels and Q1 – Q3 spreads of the scores.

## 5. Conclusions

The results of this feasibility study showed no statistically significant differences in PSS between placebo and probiotic cohorts of moderately stressed adolescents and young adults, potentially due to high within-participant variability. Salivary cortisol levels increased in the 8-week (visit 2) samples compared to baseline in both cohorts. There were no significant differences in the cortisol levels between placebo and probiotic cohorts. However, probiotic use slowed the rise in salivary cortisol levels. This is an important finding in a feasibility study that needs to be addressed in the future trial. Probiotics modulated the production of inflammatory and anti-inflammatory cytokines, including IL-6, IL-8, TNF-α, IFN-γ, and IL-10. Probiotics significantly reduced IL-1β production, a key biomarker of immune dysfunction in stressed individuals.

The results of this study cannot be generalized, as most participants were non-Hispanic Whites, who are typical representatives of urban Salt Lake City and Utah populations. Therefore, future trials need to be conducted across multiple states to represent the US population. The study did not account for stressors experienced by participants in the past and during the study. Future studies should consider collecting additional information on early-life adverse events and recent stressors experienced by participants. There was a substantial variation in the levels of study analytes, especially IL-6 and IL-8. If the main trial is conducted, the number of participants needs to be increased to confirm the observed effects and potentially achieve statistical significance. The findings of this feasibility study support further investigation of probiotic supplementation in more rigorously designed trials that account for the types of stress experienced by participants in early life and during the study and include a more ethnically diverse group of participants.

## Figures and Tables

**Table 1:** Participants’ demographic characteristics.

Categories	Placebo (*n* = 42)	Probiotics (*n* = 45)	*p* Value
*Race, ethnicity*			0.957
White, Non-Hispanic, *n* (%)	40 (95%)	39 (87%)	
White, Hispanic, *n* (%)	2 (5%)	5 (11%)	
Others, *n* (%)	NA	1 (2%)	
*Gender*			0.827
Male, *n* (%)	18 (43%)	21 (47%)	
Female, *n* (%)	24 (57%)	24 (53%)	
*Age* (Mean ± SD)	14.7 ± 2.0	15.2 ± 2.0	0.388
*PSS at pre-screening*	20.1 ± 3.3	19.6 ± 3.4	

**Table 2:** Summary of study activities.

Activity	Salivary Sample Collection	Probiotic Use	Office Visit 1	Office Visit 2	Office Visit 3
Day 1–8 after eligibility determination and consent			X		
The morning after visit 1	X				
The next day after visit 1		X			
1–2 days before visit 2	X	X			
8 weeks after visit 1		X		X	
1–2 days before visit 3	X	X			
12 weeks after visit 1		X			X

## Data Availability

The original contributions presented in this study are included in the article. Further inquiries can be directed to the corresponding author.

## Abbreviations

The following abbreviations are used in this manuscript:

HPA	hypothalamic–pituitary–adrenal
IL	interleukin
IFN	interferon
NIH	National Institutes of Health
PSS	Perceived Stress Score
TNF	tumor necrosis factor
